# Primary Angiosarcoma of Breast: Surgery Alone Versus Chemotherapy and/or Radiotherapy—A Systematic Review and Meta-Analysis

**DOI:** 10.3390/cancers18040705

**Published:** 2026-02-21

**Authors:** Konstantinos Skarentzos, Anastasia Kourtesa, Abraham Pouliakis, Menelaos G. Samaras, Andrea Palicelli, Maurizio Zizzo, Giuseppe Broggi, Serena Salzano, Rosario Caltabiano, Magda Zanelli, Nektarios I. Koufopoulos

**Affiliations:** 1 Second Department of Pathology, Medical School, National and Kapodistrian University of Athens, Attikon University Hospital, 12462 Athens, Greece; apouliak@med.uoa.gr (A.P.); mgsamaras@med.uoa.gr (M.G.S.); nkoufo@med.uoa.gr (N.I.K.); 2 Fourth Department of Internal Medicine, Medical School, National and Kapodistrian University of Athens, Attikon University Hospital, 12462 Athens, Greece; ankourtesa@med.uoa.gr; 3 Pathology Unit, Azienda USL-IRCCS di Reggio Emilia, 42123 Reggio Emilia, Italy; andrea.palicelli@ausl.re.it; 4 Surgical Oncology Unit, Azienda USL-IRCCS di Reggio Emilia, 42122 Reggio Emilia, Italy; maurizio.zizzo@ausl.re.it; 5 Department of Medical and Surgical Sciences and Advanced Technologies “G.F. Ingrassia”, Anatomic Pathology, University of Catania, 95123 Catania, Italy; giuseppe.broggi@phd.unict.it (G.B.); 1000053265@studium.unict.it (S.S.); rosario.caltabiano@unict.it (R.C.)

**Keywords:** primary angiosarcoma of the breast, PAB, systematic review, meta-analysis, therapy, treatment

## Abstract

Background: Primary angiosarcoma of the breast (PAB) is a rare malignancy with no standardized treatment protocol. Objective: To quantify the impact of tumor grade on overall survival (OS) and evaluate the association of adjuvant chemotherapy and radiotherapy with survival in primary angiosarcoma of the breast (PAB). Methods: We systematically searched PubMed, Scopus, and Cochrane Library until 27 June 2025. Two reviewers independently screened studies, extracted data, and assessed risk of bias using the Newcastle-Ottawa Scale. Kaplan–Meier curve digitization was used to reconstruct individual patient data. Random-effects meta-analysis was performed for grade comparisons and therapy associations. Results: Eleven studies (436 patients) were included. Meta-analysis of six studies showed increasing tumor grade significantly predicted mortality, with homogeneous hazard ratios (HRs) of 1.2 (Grade 2 vs. 1) and 3.7 (Grade 3 vs. 1). Analysis of five studies revealed that adjuvant chemotherapy was associated with significantly improved survival (HR = 0.11, 95% CI: 0.02–0.45), while radiotherapy showed no benefit (*p* = 0.96). Included studies demonstrated low-moderate risk of bias. Conclusions: This first quantitative synthesis establishes histologic grade as a paramount prognostic factor in PAB and shows a strong association between adjuvant chemotherapy and survival benefit. These findings provide crucial evidence for risk stratification and support considering chemotherapy in multimodal treatment for this rare disease.

## Simple Summary

Primary angiosarcoma of the breast is an exceptionally rare and aggressive malignancy with no established treatment guidelines, creating significant uncertainty in clinical management. To address this, we conducted a systematic review and meta-analysis to quantify key prognostic and treatment factors. Our findings robustly establish that the histologic grade of the tumor is a paramount determinant of patient survival. Furthermore, this quantitative synthesis demonstrates a strong association between the use of adjuvant chemotherapy and a significant improvement in survival outcomes, whereas adjuvant radiotherapy did not show a comparable benefit. These results provide crucial evidence to guide risk stratification and inform the use of multimodal therapy for this challenging disease.

## 1. Introduction

Primary angiosarcoma of the breast (PAB) is a remarkably rare and devastating malignancy of vascular origin, accounting for less than 0.05% of all primary breast malignancies [1]. Arising from the endothelial lining of blood or lymphatic vessels, PAB stands in stark contrast to epithelial breast cancers, both in its biology and its clinical behavior. It typically presents in a younger demographic of women and is notorious for its insidious onset, often without a palpable mass on mammography, which frequently leads to delayed diagnosis [2]. The aggressive nature of PAB is underscored by its high propensity for local recurrence and early hematogenous metastasis, culminating in a historically poor overall prognosis compared to more common breast carcinomas.

The clinical management of PAB is fraught with challenges, primarily stemming from its extreme rarity. The absence of large, prospective randomized controlled trials means that treatment protocols are not standardized and are largely derived from retrospective, single-institution series and expert opinion [2]. The foundational treatment for localized disease is surgical resection with the goal of achieving wide negative margins. However, due to the infiltrative growth pattern of angiosarcoma, local recurrence rates remain high even after complete resection. This has led to the widespread adoption of multimodal therapy, incorporating adjuvant chemotherapy and/or radiotherapy in an attempt to improve local and distant disease control. Despite these efforts, the precise benefit and optimal application of these adjuvant modalities continue to be a significant area of controversy and uncertainty within the field.

A critical component in the pathological assessment and risk stratification of PAB is histologic grading. The most commonly applied system, established by Donnell et al., is a three-tiered framework [3]. In this system, Grade 1 (low-grade) lesions are well-differentiated and resemble normal vascular structures, whereas Grade 3 (high-grade) tumors are characterized by poorly formed vascular channels, solid spindle cell foci, extensive necrosis, and a high mitotic index. While it is widely accepted that higher tumor grade correlates with more aggressive clinical behavior, the existing literature is composed of small, heterogeneous studies. Consequently, the exact hazard ratio associated with an increase in grade—a quantitative measure crucial for individualized prognosis and potential guidance of adjuvant therapy—has not been robustly established through meta-analysis.

The profound clinical challenge posed by PAB is exacerbated by the absence of reliable predictors to guide the use of adjuvant therapies. While complete surgical excision is universally accepted as the initial treatment, the decision to recommend additional chemotherapy or radiotherapy remains one of the most significant dilemmas in its management. This uncertainty stems from the inability to accurately identify which patients are at the highest risk for systemic recurrence and would therefore derive the greatest potential benefit from cytotoxic chemotherapy, with its associated morbidity. Similarly, the role of radiotherapy in reducing the risk of local recurrence, particularly after breast-conserving surgery or in cases with close margins, is not well-defined by high-quality evidence. Current clinical practice is thus characterized by considerable heterogeneity, often reflecting institutional preferences and individual clinician experience rather than a standardized, evidence-based algorithm. This lack of consensus can lead to both overtreatment of patients with indolent, low-grade disease and undertreatment of those with aggressive, high-grade tumors, ultimately compromising overall survival and quality of life. Clarifying not only the absolute prognostic value of tumor grade but also its potential interaction with treatment efficacy is a critical unmet need. A quantitative understanding of whether the survival benefit of adjuvant therapy (chemotherapy or radiotherapy) is concentrated within specific grade sub-groups would represent a major advance, allowing for more personalized and effective treatment strategies. Although previous comprehensive reviews have successfully summarized the epidemiological, clinical, and pathological characteristics of PAB, the literature currently lacks a quantitative synthesis of survival outcomes [4].

To address this critical gap, we conducted a systematic review and meta-analysis with two primary objectives. First, we aimed to quantitatively evaluate the impact of histologic tumor grade on overall survival (OS) in patients with PAB by pooling hazard ratios from available studies. Second, we sought to analyze the association between adjuvant therapies—specifically chemotherapy and radiotherapy—and survival outcomes. To overcome the common limitation of aggregated data in rare diseases, we employed advanced methodology, including the digitization and reconstruction of individual patient data from published Kaplan–Meier curves [5]. This approach allows for a novel and statistically powerful synthesis of the existing evidence, providing clinicians with a clearer, data-driven understanding of prognostic factors and treatment outcomes in this challenging disease.

## 2. Materials and Methods

### 2.1. Eligibility Criteria

This systematic review was conducted in accordance with the PRISMA (Preferred Reporting Items for Systematic Reviews and Meta-Analyses) guidelines and was registered prospectively with PROSPERO on 9 July 2025 (Registration ID: CRD420251079477).

The PICO (Population, Intervention, Comparator, Outcome) framework was used to define the study scope. The Population consisted of patients diagnosed with primary angiosarcoma of the breast. Patients with secondary breast angiosarcoma (e.g., following radiotherapy) were excluded. Eligible Interventions included surgery alone, surgery with adjuvant chemotherapy, surgery with adjuvant radiotherapy, or a combination of all three modalities (surgery, chemotherapy, and radiotherapy). The Comparators involved comparisons between patient groups receiving different treatment regimens or with different tumor grades. Studies were included if they reported survival outcomes (Outcomes) for these patient groups.

The exclusion criteria were as follows: case series with fewer than five patients; non-peer-reviewed articles; case reports; all types of review articles (e.g., systematic, rapid, umbrella reviews); studies utilizing large databases to prevent potential biases such as patient overrepresentation; studies with a mean or median follow-up duration of less than two years; and articles not published in the English language.

### 2.2. Information Sources, Search Strategy and Selection Process

A systematic search was performed across three electronic databases: PubMed, Scopus, and the Cochrane Library. The search was conducted from database inception until 27 June 2025, using the algorithm: “primary AND angiosarcoma AND breast”.

After removing duplicates, two independent reviewers (A.K. and K.S.), blinded to each other’s decisions, screened the records for eligibility. The screening process consisted of two phases:

1. Title and Abstract Screening: Articles were initially assessed based on their titles and abstracts against the predefined eligibility criteria.
2. Full-Text Screening: The full text of studies that passed the initial screen was retrieved. The same two reviewers then independently evaluated these articles in full text against the inclusion and exclusion criteria.

At both stages, any discrepancies between the reviewers were resolved through discussion until a consensus was reached. For the full-text screening stage, reasons for exclusion were systematically recorded. An additional manual search of the reference lists of all included studies was performed to identify any further relevant articles (a process known as “snowballing”).

### 2.3. Data Collection Process and Data Items

Data extraction was performed independently by the same two authors (A.K. and K.S.) using a standardized form. Any discrepancies in the extracted data were resolved through consensus discussion. The following data were systematically extracted from all included studies:

- Study identification: first author’s name, year of publication, country of origin, and participating hospital.
- Study characteristics: study timeline (period of data collection).
- Patient demographics: age and gender.
- Tumor characteristics: size and histologic grade.
- Treatment data: primary intervention (surgery) and adjuvant therapies (chemotherapy, radiotherapy).
- Outcomes: follow-up duration and survival data stratified by different treatment groups and tumor grades.

### 2.4. Quality Assessment

Two review authors (A.K. and K.S.), blinded to each other’s decisions, independently assessed the methodological quality of the included studies using the Newcastle–Ottawa Scale (NOS) [6]. NOS is a validated tool designed for assessing the quality of non-randomized studies. It judges studies based on three domains: selection of study groups, comparability of groups, and assessment of the outcome or exposure. The tool comprises a total of eight items, with a star system used to denote quality.

### 2.5. Synthesis Methods

The meta-analysis was conducted using the R statistical computing language (version 4.4.0) [7] and the meta package [8,9]. Since detailed individual patient data were not available from the primary studies, Kaplan–Meier (KM) curves presented in the published articles were digitized to extract numerical data for analysis. This was performed using the Web Plot Digitizer platform (https://automeris.io/WebPlotDigitizer/, accessed on 30 July 2025) to calibrate the figures and extract coordinates for overall survival (OS) and progression-free survival (PFS) over time. Time units were standardized to months for all studies, converting from days where necessary. The digitized data were then used to reconstruct KM curves within the R environment. This process enabled the estimation of survival probabilities over time and the calculation of hazard ratios (HRs) for specific grade comparisons (e.g., Grade 1 vs. Grade 2) for each individual study. For the study by Merino et al. [10], where detailed time-to-event data were provided in the text, KM curves were first reconstructed and then digitized using the same methodology to ensure consistency.

Two analytical approaches were employed:

1. Aggregated Kaplan–Meier Analysis: The digitized individual patient data from all studies were pooled to generate aggregate Kaplan–Meier curves.
2. Meta-Analysis of Hazard Ratios: A formal meta-analysis was performed based on the calculated HRs from each study. Using Grade 1 as the reference, HRs were calculated for the comparisons of Grade 1 versus Grade 2 and Grade 1 versus Grade 3.

Both fixed-effect and random-effects models were fitted. The random-effects model was considered the primary method, as it accounts for potential heterogeneity between studies and is generally preferred when clinical or methodological variations are expected. Statistical heterogeneity was quantified using the I^2^ statistics metric. The results are presented using forest plots. A sensitivity analysis was not feasible due to the limited number of studies.

The meta-analysis of grade was performed exclusively on studies utilizing the three-tier grading system to ensure prognostic consistency. Studies using the four-tier system were included in descriptive summaries but not in the pooled hazard ratio analysis for grade, to avoid misclassification bias.

## 3. Results

### 3.1. Study Selection

The systematic database search identified a total of 1404 records. This comprised 591 articles from PubMed, 805 from Scopus, and 8 from the Cochrane Library, gathered from their inception until 27 June 2025. After the removal of 398 duplicate records, 1006 unique articles were screened based on their titles and abstracts. Of these, 938 studies were excluded for not meeting the eligibility criteria.

**Figure 1:**
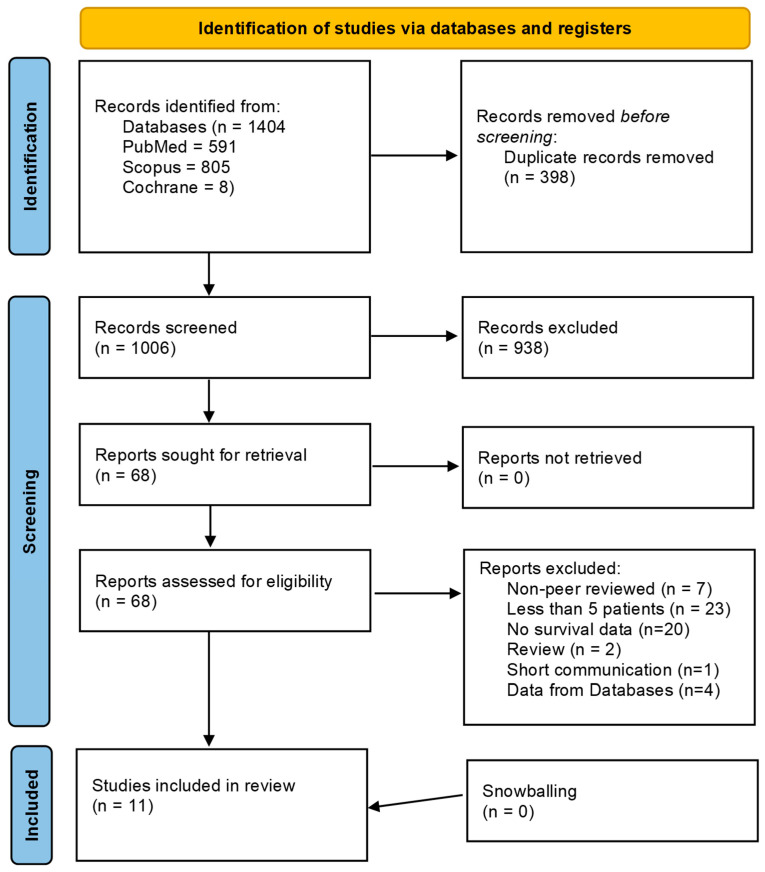
Prisma Flow Chart.

The full text of the remaining 68 articles was sought and successfully retrieved for detailed assessment. Following the full-text review, 11 studies were found to meet all inclusion criteria. The reasons for excluding the 56 full-text articles were as follows: non-peer-reviewed articles (*n* = 7), case series with fewer than five patients (*n* = 23), articles that did not report survival data stratified by tumor grade or therapy (*n* = 20), and studies based on database-derived data (*n* = 4). Furthermore, two review articles and one short communication were excluded. A manual search of the reference lists of included studies (snowballing) did not yield any additional eligible articles. The study selection process is illustrated in Figure 1 (PRISMA flow diagram).

### 3.2. Study Characteristics

A total of 436 patients from the included studies were analyzed in this systematic review [10,11,12,13,14,15,16,17,18,19]. With the exception of one study [19], which included a male patient, the study population was exclusively female.

To pool demographic and clinical characteristics, summary data were derived from the available studies. As the included studies reported median and range rather than mean and standard deviation, we applied the method by Hozo et al. [20] to estimate the mean and the range rule to estimate the standard deviation for subsequent meta-analysis.

**Figure 2:**
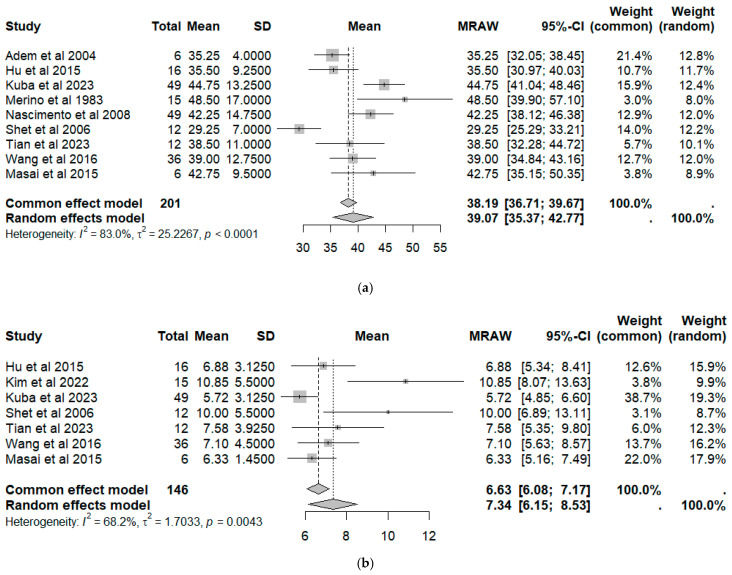
Forest plot for patients: (**a**) age; and (**b**) tumor size [1,10,11,12,13,14,16,17,18,19].

The pooled mean patient age was 38.2 (95% CI: 36.7–39.7) under the common-effect model and 39.7 (95% CI: 35.4–42.8) under the random-effects model, with considerable heterogeneity (I^2^ = 83%) (Figure 2). Similarly, the pooled mean tumor size was 6.6 mm (95% CI: 6.1–7.2) and 7.3 mm (95% CI: 6.1–8.5) for the common-effect and random-effects models, respectively, also with substantial heterogeneity (I^2^ = 68%).

Two distinct histologic grading systems were identified across the included studies. The majority of studies utilized a three-tier system [10,11,12,13,14,16,17,18,19]. In this system, Grade 1 (low grade) describes a well-differentiated tumor with relatively well-structured vascular channels. In contrast, Grade 3 (high grade) is characterized by features such as numerous mitoses, solid and spindle cell foci, papillary formation, and intralesional necrosis (Table 1) [3].

A four-tier grading system, specifically Broders’ scheme developed at the Mayo Clinic, was applied in two studies [1,15]. According to this system, a Grade 1 tumor consists of less than 25% undifferentiated cells, whereas a Grade 4 tumor consists of more than 75% undifferentiated cells (Table 2). For the purpose of the quantitative survival meta-analysis, only data from studies employing the three-tier system were pooled to maintain internal validity.

Data on therapeutic regimens were collected for the cohort. Surgery was the primary treatment received by nearly all patients. Adjuvant chemotherapy was administered to 125 patients, and 134 patients received radiotherapy. A summary of the treatment data is presented in Table 3. A descriptive summary of the specific chemotherapy regimens reported in the included studies is provided in Table 4. The most frequently documented regimens were anthracycline-based (e.g., doxorubicin), often in combination with ifosfamide. Other reported agents included taxanes (paclitaxel, docetaxel) and gemcitabine.

### 3.3. Risk of Bias

Based on the Newcastle–Ottawa Scale assessment detailed in Table 5, the overall risk of bias across the included studies was low to moderate. The majority of studies (9 out of 11) received a high-quality score of 8 or 9 stars, indicating sound methodological quality in the domains of selection, comparability, and outcome. Two studies, by Kuba et al. [13] and Shet et al. [17], achieved the maximum score of 9 stars, primarily due to superior control for confounding factors in the comparability domain. A single study, Merino et al. [10], received a slightly lower score of 7 stars, as it was marked down in the ‘Representativeness of the Exposed Cohort’ category, suggesting the study sample may not have been fully representative of the average PAB patient. Consequently, the body of evidence synthesized in this meta-analysis can be considered robust, with minimal concern that the overall conclusions are substantially compromised by systematic bias within the primary studies.

### 3.4. Results of Individual Studies

#### 3.4.1. OS in Relation to Grading

Six studies [10,11,12,13,16,19] provided sufficient details on overall survival (OS) stratified by tumor grade, making a meta-analysis feasible.

**Figure 3:**
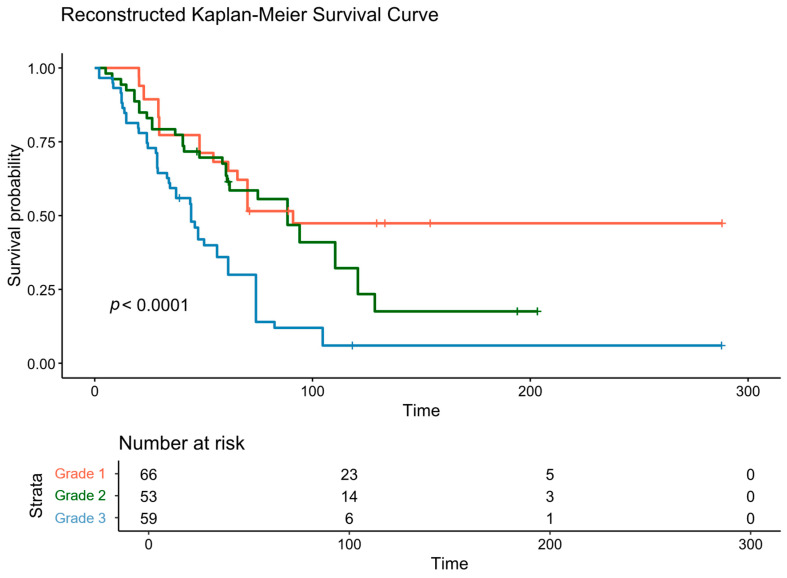
Reconstructed Kaplan–Meier curves for the overall survival in relation to the grading, along with the number of patients at risk at each time point.

The results of the meta-analysis are presented in Figure 3. Analysis of the aggregated data, comprising larger pooled populations for grades one to three (*n* = 66, *n* = 53, and *n* = 59, respectively), revealed a statistically significant difference in OS between the groups (*p* < 0.0001). The median survival times were 91.1 months (95% CI: 70.1 to not reached [inf]) for Grade 1, 88.5 months (95% CI: 60.8–129.0) for Grade 2, and 44.3 months (95% CI: 34.0–61.3) for Grade 3 patients.

**Figure 4:**
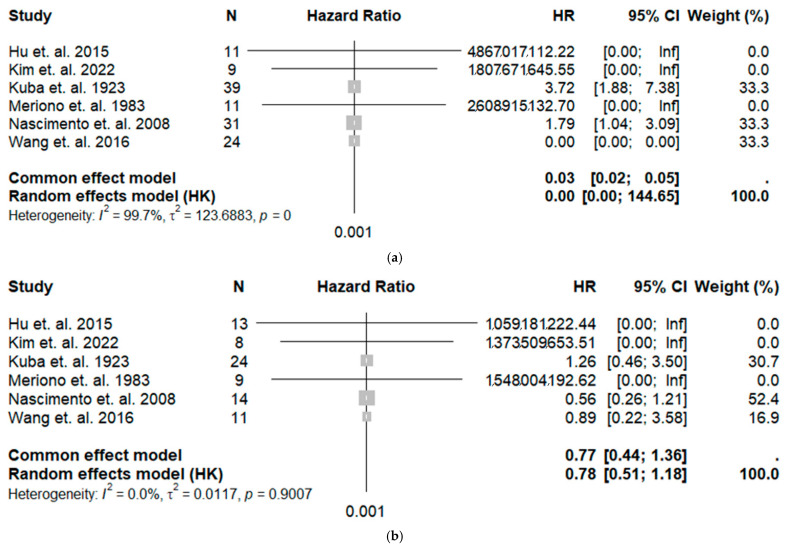
Forest plot for (**a**) the hazard ratio Grade 1 vs. Grade 2 patients (top diagram) and for (**b**) Grade 1 vs. Grade 3 patients (lower diagram), along with heterogeneity analysis and cumulative results for the common and random effects models [10,11,12,13,16,19].

The forest plots (Figure 4) display the estimated hazard ratios from the digitized survival curves. Notably, the study’s heterogeneity is rather low when comparing Grade 1 vs. Grade 2 (I^2^ = 0%), while the cumulative HR was 1.77 and 1.78 for the common and random effects models, respectively, indicating that Grade 2 patients are at higher risk when compared with Grade 1.

**Figure 5:**
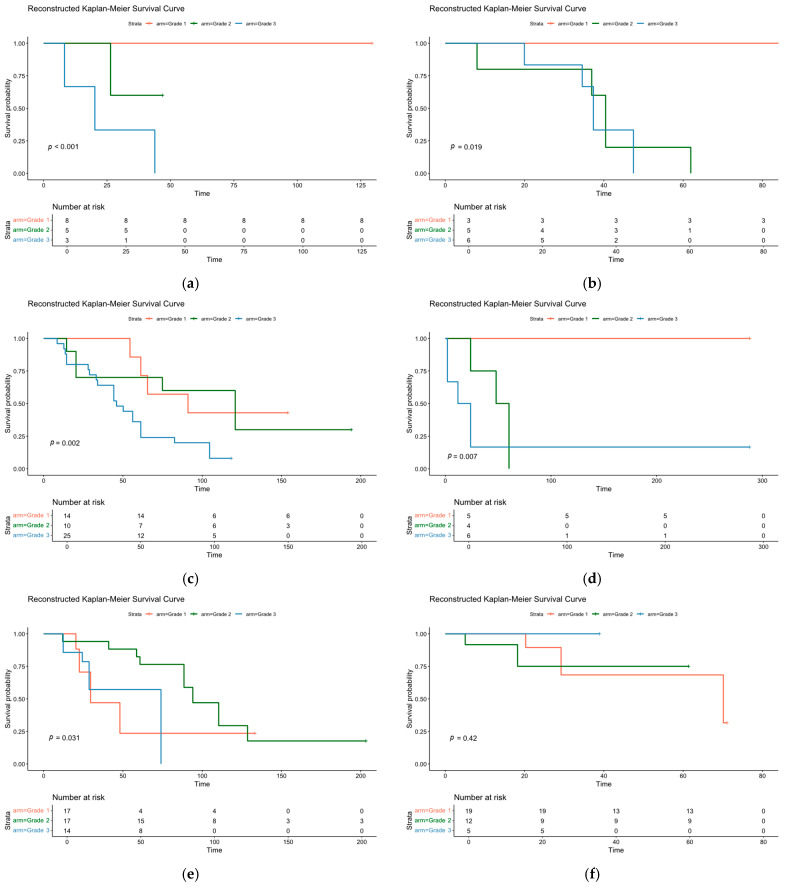
Reconstructed KM curves: from top left to lower right: (**a**) Hu et al. [11], (**b**) Kim et al. [12], (**c**) Kuba et al. [13], (**d**) Merino et al. [10], (**e**) Nascimento et al. [16] and (**f**) Wang et al. [19].

An examination of variability based on the individual study Kaplan–Meier (KM) curves (Figure 5) identified two outliers. The study by Nascimento et al. [16] was the only one in which Grade 3 patients exhibited better survival than lower-grade patients. Similarly, the study by Wang et al. [19] suggested a trend towards better OS for Grade 3, though this was not statistically significant in the reconstructed curves.

**Figure 6:**
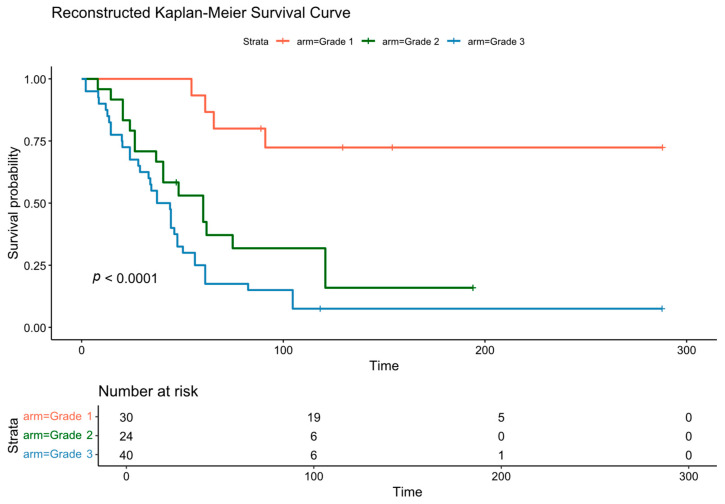
Cumulative overall survival after exclusion of the studies of Nascimento et al. [16] and Wang et al. [19].

**Figure 7:**
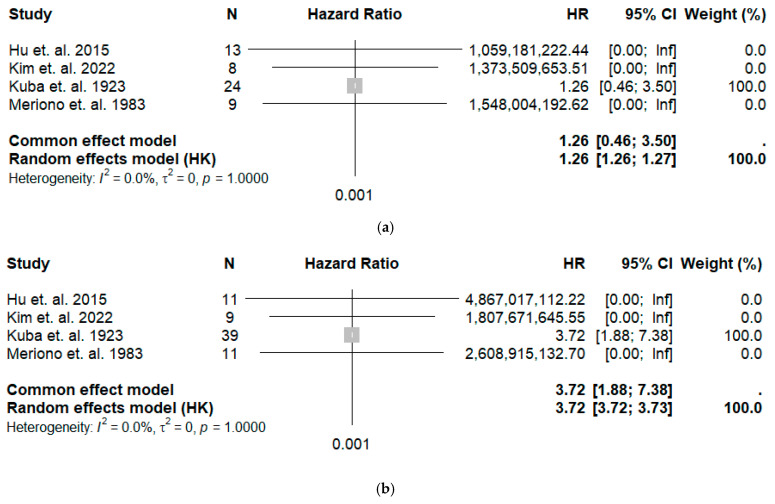
Forest plot for the hazard ratio: (**a**) Grade 1 vs. Grade 2 patients and (**b**) for Grade 1 vs. Grade 3 patients, along with heterogeneity analysis and cumulative results for the common and random effects models. The two studies of Nascimento et al. [16] and Wang et al. [19] were excluded [10,11,12,13].

Excluding these two studies yielded more stable and homogeneous results. The reconstructed KM curves for this analysis are shown in Figure 6. The restricted mean survival time (RMST) at 200 months was 120.2 months (95% CI: 101.3–139.1) for Grade 1, 93.4 months (95% CI: 74.6–110.9) for Grade 2, and 54.2 months (95% CI: 42.1–66.4) for Grade 3. The corresponding forest plots (Figure 7) showed excellent homogeneity (I^2^ = 0%). The hazard ratio was 1.2 for Grade 1 versus Grade 2 and 3.7 for Grade 1 versus Grade 3, confirming a stable, graded increase in risk.

#### 3.4.2. Raw Data Processing

**Figure 8:**
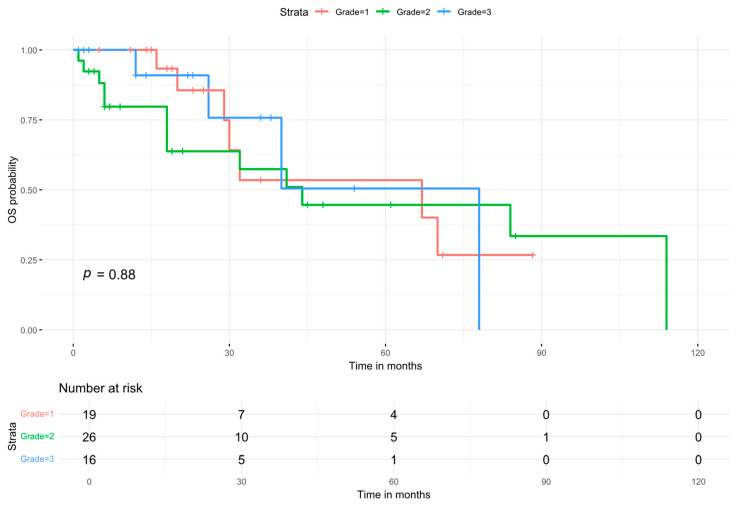
Kaplan–Meier curve for the overall survival after processing the available raw data.

Individual-level data were available from five studies [1,14,17,18,19], enabling a pooled analysis to assess the impact of tumor grade, chemotherapy, and radiotherapy. The overall survival for this pooled cohort, stratified by tumor grade, is depicted in Figure 8 with a median survival time of 44 months.

#### 3.4.3. Impact of Chemotherapy and Radiotherapy

**Figure 9:**
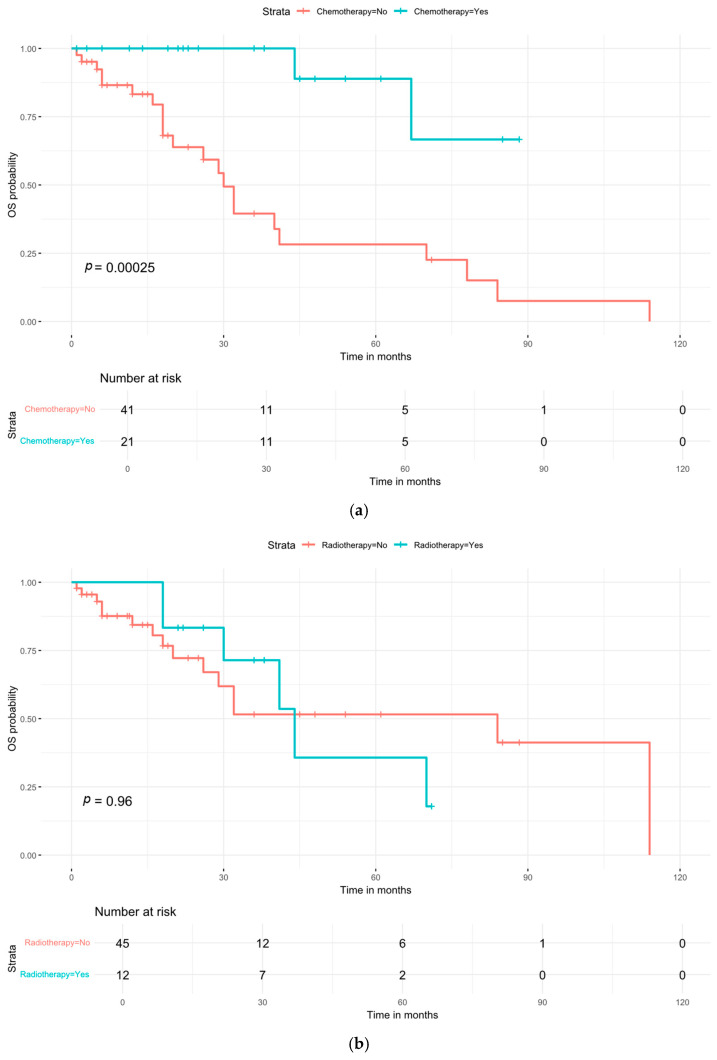
Kaplan–Meier curves for the role of: (**a**) chemotherapy and (**b**) radiotherapy after processing the available raw data.

The association between adjuvant therapies and survival from the five aforementioned studies [1,14,17,18,19] is presented in Figure 9. Patients who received chemotherapy had a significantly higher survival probability compared to those who did not (*p* = 0.0025). The hazard ratio (HR) for chemotherapy was 0.11 (95% CI: 0.02–0.45), indicating a substantial reduction in the risk of death. In contrast, radiotherapy was not associated with a survival benefit (*p* = 0.96). It is important to note that key surgical prognostic variables, most notably margin status (R0 vs. R1/R2), were not consistently reported and therefore could not be included as adjustment factors in this pooled analysis. In addition, the number of patients who received radiotherapy was relatively small, despite the inclusion of multiple studies in this analysis.

## 4. Discussion

### 4.1. General Interpretation of the Results

This systematic review and meta-analysis provides the first quantitative synthesis evaluating the roles of tumor grade and adjuvant therapy in PAB. By employing advanced data extraction techniques to pool results from multiple retrospective studies, this work directly addresses a critical evidence gap in the management of this aggressive disease. Our findings demonstrate that histologic grade serves as a powerful prognostic indicator, while suggesting potential differential effects of adjuvant chemotherapy versus radiotherapy on survival outcomes in this rare malignancy. The clear and significant difference in survival based on tumor grade confirms its critical role in risk stratification. Furthermore, the strong association with chemotherapy provides a robust, data-driven signal to inform multimodal treatment strategies, equipping clinicians with essential evidence for patient counselling and clinical decision-making.

The most robust finding from our analysis is the definitive demonstration of histologic grade as a primary determinant of survival. After accounting for study heterogeneity, we established a clear dose–response relationship: compared to Grade 1 tumors, Grade 2 tumors carried a 20% increased mortality risk, while Grade 3 tumors exhibited a 270% increased risk. This quantification provides compelling evidence that grade should be central to risk stratification in PAB. Our findings strongly support the three-tier grading system proposed by Donnell et al. [3], confirming its prognostic validity across multiple institutions. However, our results contrast with the findings of Nascimento et al. [16] and Wang et al. [19], who suggested grade was not consistently prognostic in their series, highlighting both the biological heterogeneity of PAB and the value of pooled multi-institutional data in rare diseases.

Our analysis of adjuvant therapies yielded clinically significant findings that address key controversies in PAB management. The substantial association between chemotherapy and improved survival (HR = 0.11, *p* = 0.0025) represents the strongest quantitative evidence to date supporting adjuvant chemotherapy. This finding is particularly relevant given the aggressive nature of high-grade PAB and its propensity for distant metastasis, as described by Scow et al. [2]. Notably, Wang et al. [19] also observed improved outcomes with multimodal therapy in their cohort of 36 Chinese patients, though their study was not powered to detect statistical significance for individual treatment modalities. However, as this finding is derived from non-randomized data with an inherent risk of selection bias (e.g., fitter patients or those with higher-risk disease being more likely to receive chemotherapy), it should be interpreted as a strong associative signal rather than conclusive evidence of efficacy.

It is crucial to recognize that this observed association is susceptible to significant confounding by indication. In clinical practice, adjuvant chemotherapy is preferentially offered to patients perceived to be at higher risk of recurrence, such as those with larger tumors, high-grade histology, or potentially suboptimal surgical margins. These factors are themselves powerful determinants of survival. Our analysis, based on aggregated study-level data, could not adjust for these potential confounders through multivariable modeling. Therefore, while the hazard ratio is strongly suggestive of a benefit, it should be interpreted as a robust associative signal rather than definitive proof of causal efficacy.

In contrast, we found no survival benefit associated with adjuvant radiotherapy (*p* = 0.96). This null finding should be interpreted in the context of radiotherapy’s primary goal in sarcoma management. Previous studies in breast sarcoma, including the work by Zelek et al. [4], have suggested that radiotherapy’s benefit may be primarily in local control rather than overall survival. The relatively small number of patients receiving radiotherapy in our pooled cohort, despite inclusion of multiple studies [1,14,17,18,19], limits definitive conclusions about its potential role in local control. It is important to note that the primary endpoint of this analysis was overall survival. The potential role of adjuvant radiotherapy in improving local control—a highly relevant clinical goal, particularly after breast-conserving surgery or with close margins—could not be assessed with the available data and remains an important question for future study. Consequently, while the hazard ratio is highly suggestive, this finding must be interpreted as a strong associative signal that generates a hypothesis for future prospective validation, rather than as conclusive proof of causal efficacy.

The body of evidence included in this meta-analysis was characterized by a generally low risk of bias, as assessed by the Newcastle–Ottawa Scale. This indicates that the primary studies were methodologically sound in key domains such as the selection of study groups, the ascertainment of exposures, and the assessment of outcomes. Consequently, the individual patient data synthesized herein can be considered of high quality, providing a reliable foundation for our pooled estimates of prognostic factors like tumor grade. However, a consistent limitation observed across nearly all studies was the incomplete adjustment for comparability between patient cohorts receiving different treatments. This is a fundamental challenge in retrospective sarcoma research, where treatment allocation is not randomized but is influenced by patient fitness, tumor characteristics, and institutional preferences. This inherent selection bias necessitates that our estimates of treatment efficacy, particularly for chemotherapy and radiotherapy, be interpreted with considerable caution, as residual confounding likely persists.

While our analysis focused on grade and adjuvant therapy, it is crucial to reaffirm that complete surgical resection with wide negative margins (R0 resection) remains the cornerstone of curative-intent treatment for localized PAB. The infiltrative nature of angiosarcoma, often extending beyond the visible tumor boundary, makes achieving clear margins challenging. Incompletely resected disease almost universally portends a poor prognosis. The patients included in our analysis who received adjuvant therapies presumably underwent surgery first, and the quality of that surgical resection is a critical, though often unmeasured, confounding factor. The benefit of chemotherapy observed in our study is likely contingent upon achieving maximal local control surgically. A margin-negative resection should be the non-negotiable primary objective, upon which decisions regarding adjuvant therapy are then built [21]. As reported by another important review on this topic, the choice of surgical approach requires careful consideration of tumor characteristics—including size and histologic subtype—in conjunction with patient preferences. Furthermore, the role of adjuvant radiotherapy and chemotherapy remains poorly defined, with no established consensus regarding their implementation, optimal regimens, or therapeutic efficacy [22].

The role of newer systemic agents, such as targeted therapies and immunotherapies, represents a promising area for future investigation. The molecular characterization of PAB may identify subsets of patients who could benefit from personalized treatment approaches. For instance, genomic studies in angiosarcomas from other sites have identified recurrent mutations in specific pathways, such as VEGF signaling and the PI3K/AKT/mTOR cascade, which are amenable to targeted inhibition [23,24,25]. In addition, Shon et al. demonstrated MYC amplification and overexpression in primary cutaneous angiosarcoma, highlighting a key molecular driver that may contribute to disease pathogenesis [26,27]. A comprehensive genomic analysis of 143 angiosarcoma cases identified immunotherapy-relevant biomarkers, including high tumor mutational burden and PD-L1 positivity—particularly in head and neck cases—and site-specific alterations such as MYC amplification in breast angiosarcomas [28]. Recent multiomic studies have revealed the marked complexity and heterogeneity of angiosarcomas, reinforcing the imperative for personalized therapeutic approaches [29,30].

Furthermore, the exploration of immunotherapy is particularly compelling given the vascular nature of this malignancy and the potential for enhanced immune cell access. Future research must move beyond retrospective data aggregation to prospectively validate the prognostic thresholds established in this meta-analysis. A proven framework for such an endeavor exists in the multidisciplinary, centralized approach successfully implemented for soft-tissue and visceral sarcomas across France [31]. This model, which utilizes a prospective registry to collect granular data on demographics, treatment, and outcomes for every patient presented to a specialized multidisciplinary tumor board, provides an ideal blueprint for PAB. Adopting a similar, internationally coordinated framework for PAB would directly enable the development of a universally accepted, clinically validated risk-stratification tool. By building upon this proven model, researchers could integrate tumor grade with other prognostic variables—such as tumor size, margin status, and molecular data—to create a composite risk score, thereby achieving a more precise selection of patients for adjuvant therapy than grade alone can provide.

A critical next step would be the development of a universally accepted, clinically validated risk-stratification tool that integrates tumor grade with other potential prognostic variables, such as tumor size, margin status, and patient age, to create a composite risk score for PAB patients. Building on successful multidisciplinary approaches used for soft-tissue and visceral sarcomas [31], this would allow for a more specific selection of PAB patients for adjuvant therapy than grade alone can provide. Moreover, international collaborative efforts should prioritize the creation of a prospective registry for PAB. Such a registry would not only facilitate the standardized collection of clinicopathologic data and treatment outcomes but also serve as a platform for the systematic biobanking of tumor tissue. This linked clinical and molecular data is essential for performing robust correlative studies to identify predictive biomarkers for both conventional chemotherapy and novel agents. Ultimately, the goal is to transition from a one-size-fits-all approach to a precision medicine paradigm for PAB, where treatment is tailored based on an individual tumor’s biological aggressiveness and molecular profile, thereby maximizing efficacy while minimizing unnecessary treatment toxicity for patients with this challenging disease.

### 4.2. Limitations

Our study has several important limitations that should be considered when interpreting the results. The rarity of PAB necessitated reliance on small, retrospective studies, which are susceptible to various biases. The high statistical heterogeneity observed in some analyses (I^2^ = 83% for age, I^2^ = 68% for tumor size) reflects the clinical and methodological diversity across studies, including variations in treatment protocols and the inclusion of diverse populations such as the exclusively Chinese cohort in Wang et al. [19]. The use of two different grading systems, though we attempted to reconcile them analytically, represents another source of heterogeneity. Our methodological approach of digitizing Kaplan–Meier curves, while validated by Guyot et al. [5], remains inferior to the analysis of original individual patient data. Furthermore, the potential for confounding by indication persists, as patients with more aggressive disease characteristics may have been more likely to receive adjuvant therapies.

The limitations in our study are a direct reflection of the rarity of PAB, underscoring an urgent need for a shift in the way we study this disease. Retrospective series, while valuable, have reached their limit in providing definitive answers. The establishment of an international, prospective PAB registry might be a useful next step. Such a registry would facilitate the standardized collection of data on surgical techniques, margin status, detailed chemotherapy regimens, radiotherapy fields and doses, molecular characteristics, and robust endpoints including local control, progression-free survival, and patient-reported outcomes. Such an initiative would enable the collection of large-scale, multinational, multicenter data, leading to higher-quality primary data and more definitive analyses.

### 4.3. Clinical Implications and Future Directions

Despite these limitations, our findings have important clinical implications. The strong graded relationship between tumor grade and survival supports using the three-tier system [3] for risk stratification and treatment decisions. The potential benefit of adjuvant chemotherapy warrants serious consideration, particularly for patients with high-grade tumors. The optimal chemotherapy regimen for PAB remains undefined. As illustrated in Table 4, a variety of agents, primarily anthracycline- or taxane-based, have been employed. Future prospective studies or large registry analyses should aim to compare the effectiveness of these different protocols.

Future research should focus on several key areas. First, international collaboration to establish prospective registries would help overcome the limitations of small retrospective series. Second, molecular characterization of PAB, as advocated by recent sarcoma research [21], may identify predictive biomarkers and novel therapeutic targets. Third, standardized reporting of outcomes, including local control and progression-free survival, would facilitate more comprehensive future analyses. Until more robust evidence becomes available, this quantitative synthesis represents the most comprehensive evidence base to guide the management of this challenging disease.

## 5. Conclusions

This systematic review and meta-analysis provides the first quantitative synthesis of prognostic and treatment-related survival outcomes for primary angiosarcoma of the breast (PAB). Our findings robustly confirm histologic grade as an independent prognostic factor, with a clear, graded increase in mortality risk. Furthermore, we identified a strong association between the administration of adjuvant chemotherapy and a significant survival benefit, whereas adjuvant radiotherapy was not associated with improved overall survival in our pooled analysis. These results directly address critical gaps in the evidence base for this rare malignancy. They provide clinicians with essential data to support risk-stratified decision-making and underscore the potential value of chemotherapy and provide a robust hypothesis for its benefit.

Given the inherent limitations of retrospective data, these conclusions should inform, rather than definitively dictate, clinical practice. The optimal integration of these findings requires multidisciplinary discussion, balancing the quantified risks and potential benefits for each patient. Future efforts must focus on international collaboration to establish prospective registries, validate these associations in larger cohorts, and explore the role of novel systemic agents to further improve outcomes for patients with this challenging disease.

## Figures and Tables

**Table 1:** Three-tier grading system.

Authors	Grade 1 (Low)	Grade 2 (Intermediate)	Grade 3 (High Grade)	Unidentified
Hu et al. [11]	8	5	3	0
Kim et al. [12]	3	5	6	1
Kuba et al. [13]	14	10	25	0
Merino et al. [10]	5	4	6	0
Nascimento et al. [16]	17	17	14	0
Shet et al. [17]	1	9	2	0
Tian et al. [18]	2	5	4	1
Wang et al. [19]	19	12	5	0
Masai et al. [14]	1	0	5	0
Total	70	67	70	2

**Table 2:** Four-tier grading system.

Authors	Grade 1	Grade 2	Grade 3	Grade 4	Unidentified
Adem et al. [1]	1	3	1	1	0
McClelland et al. [15]	57	40	51	25	47
Total	58	43	52	26	47

**Table 3:** Therapy.

	Surgery (n)	Chemotherapy (n)	Radiotherapy (n)
Authors	Yes-No-Unknown	Yes-No-Unknown	Yes-No-Unknown
Adem et al. [1]	6-0-0	0-6-0	1-5-0
Hu et al. [11]	16-0-0	7-9-0	5-11-0
Kim et al. [12]	15-0-0	4-11-0	8-7-0
Kuba et al. [13]	49-0-0	17-32-0	13-36-0
McClelland et al. [15]	220-0-0	65-155-0	81-139-0
Merino et al. [10]	15-0-0	3-12-0	5-10-0
Nascimento et al. [16]	46-3	8-27-14	10-26-13
Shet et al. [17]	12-0-0	0-12-0	2-10-0
Tian et al. [18]	12-0-0	10-2-0	2-10-0
Wang et al. [19]	28-0-8	10-18-8	7-21-8
Masai et al. [14]	6-0-0	1-5-0	0-6-0
Total	425-3-8	125-289-22	134-281-21

**Table 4:** Chemotherapy Regimen/Agents.

Authors	Chemotherapy Regimen/Agents
Adem et al. [1]	No chemotherapy was given
Hu et al. [11]	Cyclophosphamide + Doxorubicin
Kim et al. [12]	Adriamycin + Ifosfamide or Etoposide + Ifosfamide (for pediatric patients) followed by Paclitaxel or Adriamycin + Cyclophosphamide
Kuba et al. [13]	Gemcitabine + Docetaxel (one case explicitly mentioned; other regimens not specified)
McClelland et al. [15]	Not reported
Merino et al. [10]	Not reported
Nascimento et al. [16]	Not reported
Shet et al. [17]	No chemotherapy was given
Tian et al. [18]	Doxorubicin + Ifosfamide + Dacarvazine +/− Mesna or Taxane with Gemcitabine/Doxorubicin
Wang et al. [19]	Not reported
Masai et al. [14]	Adriamycin + Ifosfamide + Dacarbazine + modified Mesna

**Table 5:** Risk of Bias/Quality Assessment.

Authors	Representativeness of the Exposed Cohort (0–1)	Selection of the Non-Exposed Cohort (0–1)	Ascertainment of Exposure (0–1)	Demonstration That Outcome of Interest Was Not Present at Start of Study (0–1)	Comparability of Cohorts on the Basis of the Design or Analysis (0–2)	Assessment of Outcome (0–1)	Was Follow-Up Long Enough for Outcomes to Occur (0–1)	Adequacy of Follow-Up of Cohorts (0–1)	Total Score (0–9)
Tian et al. [18]	1	1	1	1	1	1	1	1	8
Kim et al. [12]	1	1	1	1	1	1	1	1	8
Hu et al. [11]	1	1	1	1	1	1	1	1	8
Kuba et al. [13]	1	1	1	1	2	1	1	1	9
Merino et al. [10]	0	1	1	1	1	1	1	1	7
Nascimento et al. [16]	1	1	1	1	1	1	1	1	8
Adem et al. [1]	1	1	1	1	1	1	1	1	8
Wang et al. [19]	1	1	1	1	1	1	1	1	8
McClelland et al. [15]	1	1	1	1	1	1	1	1	8
Masai et al. [14]	1	1	1	1	1	1	1	1	8
Shet et al. [17]	1	1	1	1	2	1	1	1	9

## Data Availability

The data presented in this study are available on request from the corresponding author.

## Abbreviations

The following abbreviations are used in this manuscript:

CI	Confidence Interval
HR	Hazard Ratio
I^2^	I-squared statistic
KM	Kaplan–Meier
NOS	Newcastle-Ottawa Scale
OS	Overall Survival
PAB	Primary Angiosarcoma of the Breast
PFS	Progression-Free Survival
PICO	Population, Intervention, Comparator, Outcome
PI3K/AKT/mTOR	Phosphoinositide 3-kinase/Protein Kinase B/Mammalian Target of Rapamcin
PRISMA	Preferred Reporting Items for Systematic Reviews and Meta-Analyses
PROSPERO	International Prospective Register of Systematic Reviews
R0	Resection with microscopically negative margins
RMST	Restricted Mean Survival Time
VEGF	Vascular Endothelial Growth Factor
